# Rationally engineered advances in cancer research

**DOI:** 10.1063/1.5056176

**Published:** 2018-09-26

**Authors:** Adam J. Engler, Dennis E. Discher

**Affiliations:** 1 Department of Bioengineering, University of California, San Diego, La Jolla, California 92093, USA; 2 Sanford Consortium for Regenerative Medicine, La Jolla, California 92037, USA; 3 Biophysical Engineering Labs, University of Pennsylvania, Philadelphia, Pennsylvania 19104, USA

## Abstract

The physical and engineering sciences have much to offer in understanding, diagnosing, and even treating cancer. Microfluidics, imaging, materials, and diverse measurement devices are all helping to shift paradigms of tumorigenesis and dissemination. Using materials and micro-probes of elasticity, for example, epithelia have been shown to transform into mesenchymal cells when the elasticity of adjacent tissue increases. Approaches common in engineering science enable such discoveries, and further application of such tools and principles will likely improve existing cancer models *in vivo* and also create better models for high throughput analyses *in vitro*. As profiled in this special topic issue composed of more than a dozen manuscripts, opportunities abound for the creativity and analytics of engineering and the physical sciences to make advances in and against cancer.

## INTRODUCTION

The complex, heterogeneous structures of tumors and their propensity to invade other tissues of the body have significantly slowed the potential progress toward understanding cancer and its origins and treatments. Engineering approaches, on the other hand, take inherently complex systems and reduce them to their constituent parts, properties, and processes. A tumor could include not only cells within the tumor but also the surrounding extracellular matrix (ECM) that anchors the tumor, stromal fibroblasts that secrete proteins for and support the tumor, and the various fluids that flow into and out of the tumor. Each aspect requires a careful study, and the manuscripts published in this special issue are testament to that general approach. They deepen understanding by advanced imaging or by building simple systems to focus on a microenvironment property, such as ECM that is stiffer,^1^ more dense,^2^ crosslinked,^3^ aligned,^1^ or less porous.^3^ The efforts profiled here investigate or summarize our knowledge of tumors in four ways: (1) some clarify how intratumor or stromal fluid^4,5^ or blood flow^6^ drives tumor behavior, (2) others develop model *in vitro* systems to understand^7–10^ or measure^11^ tumor behavior, (3) still others examine how cancer cells sense changes in their niche^12^ and how that drive behaviors,^13,14^ and (4) a few are more integrative with computational simulations of complex processes in cancer.^15–17^

## FLUID FLUX WITHIN AND AROUND TUMORS

Because we are about 70% water, our tissues are constantly transporting fluids—and not just blood. Almost two decades ago, engineers were beginning to create complex mathematical models of such flows in tumors^18^ but not until recently have non-invasive imaging methods been able to observe these flows *in vivo*. Kingsmore and co-workers^4^ used dynamic contrast-enhanced magnetic resonance imaging (MRI) and a training model to validate *in situ* that their dynamic contrast enhancement and analytical processing could yield accurate velocity vectors within a glioblastoma multiforme (GBM) xenograft model. Heterogeneous fluid flows within and around tumors suggest further refinements for mathematical models and correlations with drug treatment.^19^ Similar dynamics with solid shear stress, among other mechanical inputs, could also affect solid tumors. Mehta and co-workers^5^ summarized how both fluid shear, in particular ascites flow, through the ovary and solid shear from relative movements within the body could impact tumor behavior. Mehta *et al.* surmised that bioreactors and other fluid shear devices can greatly improve our understanding of such forces, thus highlighting how engineering sciences might illuminate some aspects of complex cancer processes.

## CANCER-IN-A-DISH

Reducing a system's complexity through engineering methods can help advance cancer biology in terms of mechanistic understanding or perhaps with a goal of exploiting a cell's properties as a means of separation. Both approaches are highlighted in this special issue. For example, it can be difficult in normally complex tumors to identify how clinically relevant doses of radiation affect cells and their matrix,^20^ but as Reinhart-King and coworkers showed, ionizing radiation reduces collagen matrix stiffness without concomitant changes to matrix porosity or architecture. Non-irradiated cancer cells then show reduced adhesion, spreading, and migration when plated onto the irradiated matrix, suggesting that stiffness changes may affect tissue mechanics. Cell arrangement can also be a key feature of a reductionist system. Mammary epithelial cells form sheets in 2D and hollow acinar structures in 3D; their mechano-sensing is also dramatically altered between these states^1,21^ as cell-ECM and cell-cell cues balance each other. Tanner^10^ highlighted how hydrogels and other systems can be modified to probe mechanobiological questions in the brain. Kumar and coworkers^9^ further demonstrated this principal for GBM by fabricating a 3D hyaluronic acid (HA) hydrogel system with channels to embed cells and mimic vasculature. They observed invasion of the parenchymal-like HA or collagen I hydrogel and subsequent penetration of the vascular mimic, whereby cells migrate faster on collagen. Similarly, Fleszar and coworkers^7^ probed the effects of the matrix composition on fallopian tube epithelium in a 3D system with a built-in lumen. As with Kumar and coworkers, Fleszar found that specific collagen types along with the presence of fluid shear influence epithelium behavior, including migration and invasion into surrounding stroma which is relevant to cortical inclusion cysts in ovarian cancers. Engineering approaches can thus create devices in which to test reductionist hypotheses that would be exceedingly difficult to evaluate in a controlled manner *in vivo*.

The bioengineering alternative to studying cell behaviors in a reductionist environment is to exploit a cell's properties as a means of separation. Over the past decade, systems have been developed to separate cancer cells from solid tissues and blood. While most associate cancer with solid tumors, cancer cells must hijack the vasculature to spread to distant tissues,^22^ and this has provided a convenient entry point for many separation methods; engineers have used fluidics to centrifuge larger cancer cells from buffers and blood.^23^ Fluidics and optics together allow the user to stretch^24,25^ or squeeze cells,^26^ determine their deformability, and then make sorting decisions; cancer cells generally are softer than their surrounding counterparts, and use of this principle to sort cancer cells is sometimes referred to as deformation cytometry (DC). Recent efforts have included enhanced throughput to make real-time DC^27^ and as highlighted here by Ahmmed and coworkers,^11^ multiplexed sorting can decrease the sorting times by increasing the channel number on the chip. While some of the above systems are sufficiently large to prevent clogging, a multiplexed sorting chip can help ensure a continuous sort process.

## I'VE GOT A FEELIN': SENSING AND RESPONSE IN CANCER

A third area that bioengineering research has “metastasized” to is understanding to what extent and why cells respond to the cues in *in vitro* systems. Subfields of mechanobiology and mechanotransduction often address these questions, and three examples of such work in this special issue highlight distinct signaling mechanisms. First, it is important to establish if a cell behavior in an engineered system is guided by single or multicellular responses; such is the focus of the work by Gligorijevic and co-workers^14^ where the balance between contact guidance and chemotactic cues was examined together with the cell cycle and proliferation. Cells might “decide” to migrate singly through constrictions, even going so far as to rupture their nuclei.^28,29^ Heureaux-Torres and coworkers^12^ presented data in this issue, suggesting that signaling from the mechanosensitive channel of large conductance (MscL) may serve as a “go”-“no go” switch; MscL expressing cancer cells migrated at the same velocity as their non-expressing counterparts, but Heureaux-Torres observed that MscL cells more frequently get “stuck” at the entrance of constricting channels. They fail to crawl into a channel at higher rates, perhaps sensing that the constriction is too excessive. In migrating through tumor stroma, cancer cells have been observed to fuse with stromal cells, and Chitwood and coworkers^13^ discovered that hybrid cells form spontaneously and at a significantly higher rate in metastases. This could add genetic diversity and also provide transcripts and proteins from primary stroma to condition the distant niche at a metastatic site. In all of these examples, it is critical to note that engineered systems, whether a reductionist *in vitro* mimic or a specific reporter probe engineered to fluoresce under specific conditions, were critical in identifying these mechanisms.

## COMPUTING COMPLEXITY, INCLUDING MUTATIONS

As cells migrate and find new niches, they must integrate a plethora of signals along their path. Reductionist approaches in this instance can often oversimplify the system and miss important emergent behaviors. *In silico* approaches using computational modeling can take individual parameters known to be influential and test them in a large combinatorial matrix to establish, for example, that in 2D, stiffness, ligand density, and ligand composition affect metastatic cancer cell migration, but in 3D, tight pores necessitate migration mediated by matrix degradation.^30^ While migration is often examined individually, cancer cells can also exhibit coordinated migration.^31^ In this issue, Sun and coworkers^16^ developed a collective migration model for confluent epithelia based on vertex modeling, i.e., where cancer cells are treated as polygons with shared vertices and edges. Cells within the layer experience passive, frictional, and contractile forces from cell-cell and cell-ECM contacts, and when arranged as a sheet, individual cancer cells undergo periodic migration during continuous migration of the sheet or streaming behavior, i.e., rotation of the sheet. Differences in this behavior occur as a function of density, contractility, and persistence. Along with migration, epithelia make equally complex decisions to undergo epithelial-to-mesenchymal transition (EMT) or not; numerous overlapping transcription factors govern expression of the genes that control this process, and these factors are activated by a number of external cues.^32–34^ Also in this issue, Jolly and coworkers^15^ examined the pathways that lead to EMT, identifying a series of feedback loops that enable epithelial cells to maintain a stable, hybrid phenotype with both epithelial and mesenchymal characteristics. Their model also indicates ESRP1 as a crucial signaling node that when knocked down in a cancer line leads from this stable hybrid state into EMT; in patients, it correlates with poor prognosis.

Cancer only arises from mutations, and computational approaches can also usefully mine big data that are rapidly accumulating from whole-genome sequencing (WGS). The considerable engineering advances that profoundly reduced the costs of sequencing are now producing tera-bytes of such data for an increasing number of patients—as was the vision of physicists and engineers in the Department of Energy who first conceived of the Human Genome Project decades ago in order to sequence DNA for radiation-induced mutations.^35^ In this issue, Alter and coworkers^17^ analyzed patient-matched astrocytomas and non-malignant tissues using generalized singular value decomposition and uncovered patterns in genes within Notch, Ras, and Shh pathways. Compared to age or tumor grade, the patterns provide more accurate predictors of recurrence free survival and response to chemotherapy and radiation therapy.

In summary, this special issue provides an opportunity for readers to experience all the ways that bioengineering can be applied to cancer: to precisely measure individual components of a system to take an inherently complex system and reduce it to constituent parts to examine key features or to model complexity. While conventional biological approaches have brought tremendous advances to cancer diagnostics and treatments over the last century, it is the opportunity that engineering analysis affords as highlighted here that may very well propel us into the next century of cancer breakthroughs.
